# Splenectomy Promotes Macrophage Polarization in a Mouse Model of Concanavalin A- (ConA-) Induced Liver Fibrosis

**DOI:** 10.1155/2019/5756189

**Published:** 2019-01-06

**Authors:** Yongjuan Wang, Xiaopei Guo, Guohui Jiao, Lili Luo, Lu Zhou, Jie Zhang, Bangmao Wang

**Affiliations:** ^1^Department of Gastroenterology and Hepatology, Tianjin Medical University, General Hospital, Tianjin, China; ^2^Department of Gastroenterology and Hepatology, The Second Affiliated Hospital of Hebei Medical University, Hebei, China; ^3^Department of Geriatric Medicine, Tianjin Medical University, General Hospital, Tianjin, China

## Abstract

**Background:**

Splenectomy can improve liver function and survival in patients with autoimmune hepatitis (AIH) and liver cirrhosis. We investigated the underlying mechanism in a mouse model of concanavalin A- (ConA-) induced liver fibrosis.

**Methods:**

We used ConA to induce immune liver fibrosis in BALB/c mice. Splenectomy was performed alone or with the administration of dexamethasone (DEX). Changes in blood and liver tissues were evaluated.

**Results:**

Mice treated with ConA for 7 weeks developed advanced liver fibrosis, while splenectomy suppressed liver fibrosis. Although the populations of macrophages/monocytes and M1 macrophages decreased after splenectomy, the inflammatory factors associated with M2 macrophages increased after splenectomy. Furthermore, the population of circulating CD11b^+^Ly6C^high^ myeloid-derived suppressor cells (MDSCs) increased after splenectomy. After ConA treatment, elevated levels of activated and total NF-kBp65/p50 combined with DNA were observed in hepatic tissues. In contrast, the levels of NF-*κ*B p65/p50 decreased after splenectomy.

**Conclusions:**

Splenectomy may promote the polarization of CD11b^+^Ly6C^high^ MDSCs and the differentiation of M2 macrophages while restricting the level of NF-*κ*B p65-p50 heterodimers. These factors may suppress the progression of liver fibrosis.

## 1. Introduction

Autoimmune hepatitis (AIH) is characterized by the infiltration of mononuclear cells into the liver which, together with elevated levels of gamma globulins and autoantibodies, can induce fibrosis and cirrhosis [1]. Liver cirrhosis frequently causes portal hypertension and splenomegaly and eventually liver failure. Recent studies in various clinical settings have indicated that splenectomy can lead to improvements in both liver function parameters and thrombocytopenia [2, 3]. Moreover, a study by Maruoka et al. demonstrated that splenectomy could treat corticosteroid insufficiency in mice and increased the duration of positive effects from dexamethasone (DEX) [4]. Nonetheless, the mechanism behind this phenomenon remained unknown.

Kupffer cells (KCs) are a subset of highly heterogeneous macrophages that can be stratified into types M1 and M2 [5, 6]. Bacterial lipopolysaccharide (LPS) can stimulate the differentiation of macrophages to the M1 type, which produce the inflammatory factors tumor necrosis factor- (TNF-) *α*, interleukin- (IL-) 12, IL-23, and inducible nitric oxide synthase (iNOS). In contrast, IL-4, IL-13, IL-10, and glucocorticoids can induce macrophage polarization to the M2 type, which produce cytokines such as IL-4, IL-5, and IL-10, which can inhibit inflammation while promoting blood and lymphatic vessel formation, digestion, and extracellular matrix repair [7, 8]. Therefore, macrophages and macrophage-related factors might play an essential role in the development and pathogenesis of hepatic lesions and fibrosis [9–11]. However, the polarization of M1/M2 macrophages in AIH remains unclear.

Myeloid-derived suppressor cells (MDSCs) comprise a heterogeneous population of cells that inhibit the proliferation and regular functions of T cells, suppress the cytotoxicity of NK cells, and accelerate the polarization of regulatory T cells (Tregs) in tumor-bearing hosts, all of which play key regulatory roles in tumor-related diseases [12, 13]. In mice, MDSCs are CD11b^+^Gr1^+^ myeloid cells, which can be subdivided into CD11b^+^Gr1^+^Ly6G^high^  Ly6C^low^ and CD11b^+^Gr1^+^Ly6G^low^Ly6C^high^ MDSCs. Although many reports have discussed the immunosuppressive function of MDSCs in various cancer-related diseases, the specific regulatory mechanisms of these cells in immunological liver disease remained unclear [13, 14]. Zhang et al. established a model of acute immunological liver injury by injecting BALB/c mice with concanavalin A (ConA) and found that rapamycin could promote the proliferation of CD11b^+^Gr1Ly6C^high^MDSCs, which protected against immunological hepatic injury in this model [15]. This led us to wonder whether splenectomy could inhibit liver fibrosis by promoting the polarization of MDSCs.

NF-*κ*B, a key transcription factor, is found in cell types throughout the body. This factor is a homo- or heterodimer comprising the p50 and p65 (RelA) subunits [16]. The regulation of NF-*κ*B signaling may lead to macrophage-driven inflammation in both autoimmune and hematological diseases [17, 18]. Furthermore, NF-*κ*B p50 can regulate M2 polarization in the context of LPS-induced inflammation [19]. However, the correlation between macrophage infiltration and underlying NF-*κ*B function in immune-related liver fibrosis remained unclear.

Con A-induced hepatitis is a widely accepted and successful mouse model of AIH that resembles advanced immune-related liver fibrosis in humans [20, 21]. In this study, we investigated the effects of splenectomy in a mouse model of ConA-induced liver fibrosis and determined whether MDSCs and NF-*κ*B were necessary to the protective effects of splenectomy against liver cirrhosis in these animals.

## 2. Methods

### 2.1. Animal Models

The study was approved by the Medical Ethics Committee of the General Hospital of Tianjin Medical University. All animals used experimentally were treated humanely, and all procedures were conducted according to the guidelines set forth by the Animal Care Committee of the General Hospital of Tianjin Medical University. Specific pathogen-free female BALB/c mice (7 weeks of age) were acquired from Beijing, China. A total of 24 mice were used for the experiments. The mice were randomly assigned to four groups: the control group, ConA model group, splenectomy group, and splenectomy combined with dexamethasone (DEX) group. Except mice in the control group, all mice received a weekly dose of 12.5 mg/kg body weight of ConA via tail vein injection. Splenectomy was conducted 1 day after the sixth injection. Mice in the splenectomy combined with DEX group also received an intraperitoneal injection of DEX at 1 mg/kg body weight every second day. To mimic the presence of immune damage* in vivo*, ConA treatment was maintained until death. All mice were sacrificed after 7 weeks, and blood and liver tissue samples were collected. The liver tissues were embedded in paraffin and subjected to hematoxylin and eosin (H&E) staining and Masson's trichrome staining.

### 2.2. Splenectomy

For splenectomy, the abdominal wall of the mouse was opened by making a left subcostal minimal incision under chloral hydrate. The splenic arteries and veins were ligated at the splenic hilum with a 3–0 silk suture and divided. All surgical procedures were conducted under completely sterile conditions.

### 2.3. Histological Analysis

The histological analysis procedures have already been outlined according to previous reports [22, 23]. Liver tissues were fixed in 10% buffered formalin and embedded in paraffin. Subsequently, the samples were stained with H&E and Masson's trichrome (to assess fibrosis) and evaluated under a light microscope.

### 2.4. Immunohistochemical Staining

Samples were also subjected to immunohistochemistry. A goat monoclonal antibody (mAb) specific for human CD68 (Santa Cruz Biotechnology, Dallas, TX, USA) and mouse mAb specific for human CD206 (Santa Cruz Biotechnology) were used to label macrophages and M2 macrophages. A goat mAb specific for mouse *α*-smooth muscle actin (SMA, Santa Cruz Biotechnology) was used to label *α*-SMA. The labeled sections were further incubated with a biotin-free secondary antibody. Horseradish peroxidase (Santa Cruz Biotechnology) was then applied, and the labeled tissues were developed using diaminobenzidine (Sigma, St Louis, MO, USA) followed by hematoxylin counterstaining.

### 2.5. Immunofluorescence Staining

A goat mAb specific for human CD68 and mouse mAb specific for human CD206 (both Santa Cruz Biotechnology) were used to immunofluorescently label macrophages and M2 macrophages. Goat mAbs specific for mouse F4/80 and mouse iNOS (both Abcam, Cambridge, MA, USA) were used to immunofluorescently label macrophages and M1 macrophages. The following reagents were used in all immunofluorescence experiments: Alexa 488-labeled donkey anti-rat, Alexa 647-labeled donkey anti-rabbit antibodies (Molecular Probes, Eugene, OR, USA), Alexa Fluor 568-labeled donkey anti-mouse, and Alexa Fluor 488-labeled rabbit anti-goat. DAPI was used for nuclear counterstaining.

### 2.6. Quantitative RT-PCR (Real-Time PCR)

The RT-PCR analysis was conducted as previously described [24, 25].  Table 1 lists the primers used in this experiment. The data were analyzed using SDS 2.1 software. For all target genes, the expression is represented as a “fold change” relative to the control sample (2-^△△^comparative threshold).

### 2.7. MAbs and Flow Cytometry

MAbs specific for CD11b (M1/70), F4/80 (BM8), and Ly6C were obtained from BioLegend (San Diego, CA, USA). The cells were suspended in buffer and incubated with the above antibody for 30 minutes. Subsequently, the cells were analyzed on a FACSCalibur flow cytometer (Becton Dickinson, San Jose, CA, USA) or Beckman Coulter Epics XL bench-top flow cytometer (Beckman Coulter, Brea, CA, USA). Data were analyzed using FlowJo software (TreeStar, Ashland, OR, USA). The number of cells in several different populations was calculated by multiplying the percentages of the counted targeted cells by the number of total cells.

### 2.8. Western Blotting

Polyvinylidene fluoride (PVDF) membranes were incubated overnight at 4°C with antibodies specific for NF-*κ*B p50 and NF-*κ*B p65 (Santa Cruz Biotechnology). Samples were then washed in Tris-buffered saline with Tween-20 and labeled further with a goat anti-mouse secondary antibody (LI-COR Biotechnology, Lincoln, NE, USA). Labeled protein bands were detected using an Odyssey infrared imaging system (LI-COR). Glyceraldehyde 3-phosphate dehydrogenase (GAPDH) was used as an internal reference control.

### 2.9. Electrophoretic Mobility Shift Assay (EMSA)

PCR primers were labeled with [-33P]-ATP (Perkin Elmer, Waltham, MA, USA) using T4 DNA polynucleotide kinase (New England Biolabs, Ipswich, MA, USA). Subsequently, DNA probes were created by labeling the forward primers and unlabeling the reverse primers via PCR. Two micrograms of nuclear protein were incubated with a biotin-labeled NF-*κ*B p65 binding-site DNA probe (5′-AGTTGAGGGGACTTTCCCAGGC-3′; Sigma Genosys) in buffer for 30 minutes on ice. Gels were placed on Whatman paper, vacuum-dried, and exposed to a phosphoscreen for 12 hours. Subsequently, the screen was scanned using a storm 860 PhosphorImager, and the data were analyzed using ImageQuant software (Molecular Dynamics/GE Healthcare, Amersham, UK).

### 2.10. Statistical Analysis

All numeric values are expressed as means ± standard deviations (SD). Students' t-test or the Mann–Whitney U test was used to detect the statistical significance of differences between groups. Multiple group comparisons were assessed using a one-way analysis of variance (ANOVA) combined with Bonferroni's post hoc test. A* P* value<0.05 was considered the statistical significance threshold.

## 3. Results

### 3.1. Effects of Splenectomy in a Mouse Liver Fibrosis Model

**Figure 1:**
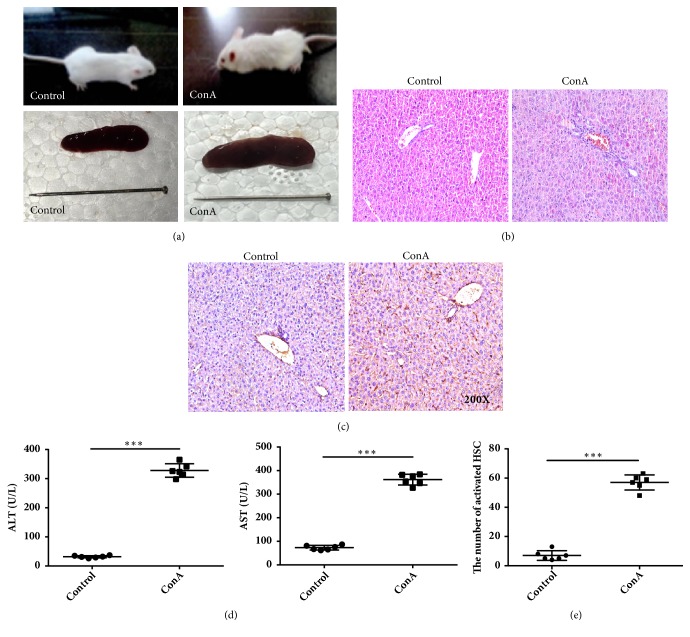
Morphological and immunohistochemical analysis of liver tissues in control and concanavalin A- (ConA-) treated mice. (a) Mice and liver in the control and ConA-treated groups. (b) Hematoxylin and eosin staining showing inflammatory cell infiltration in the portal area. (c) Immunohistochemical analysis of *α*-smooth muscle actin (SMA) expression in liver tissues (immunohistochemical stain, magnification ×200). (d) Alanine (ALT) and aspartate aminotransferase (AST) levels detected using an enzyme-linked immunosorbent assay. (e) The numbers of activated hepatic stellate cells (HSC). *∗∗∗* indicates* P* < 0.001.

Clear increases in splenic volume were observed after a 5-week course of intravenous ConA injection, and the resulting immune hepatic injury was characterized by hepatocellular necrosis, portal inflammation, mononuclear cell infiltration into the parenchyma, and sinusoidal hyperemia (Figures 1(a), 1(b), and 1(c)). These tissue changes were concomitant with significant increases in the serum levels of aspartate aminotransferase (AST) and alanine aminotransferase (ALT (*P*<0.05) (Figure 1(d)). Most importantly, immunohistochemical staining of *α*-SMA revealed a marked and significant increase in the number of activated hepatic stellate cells (*P*<0.05) (Figure 1(e)).

### 3.2. Splenectomy Attenuated ConA-Induced Liver Fibrosis

**Figure 2:**
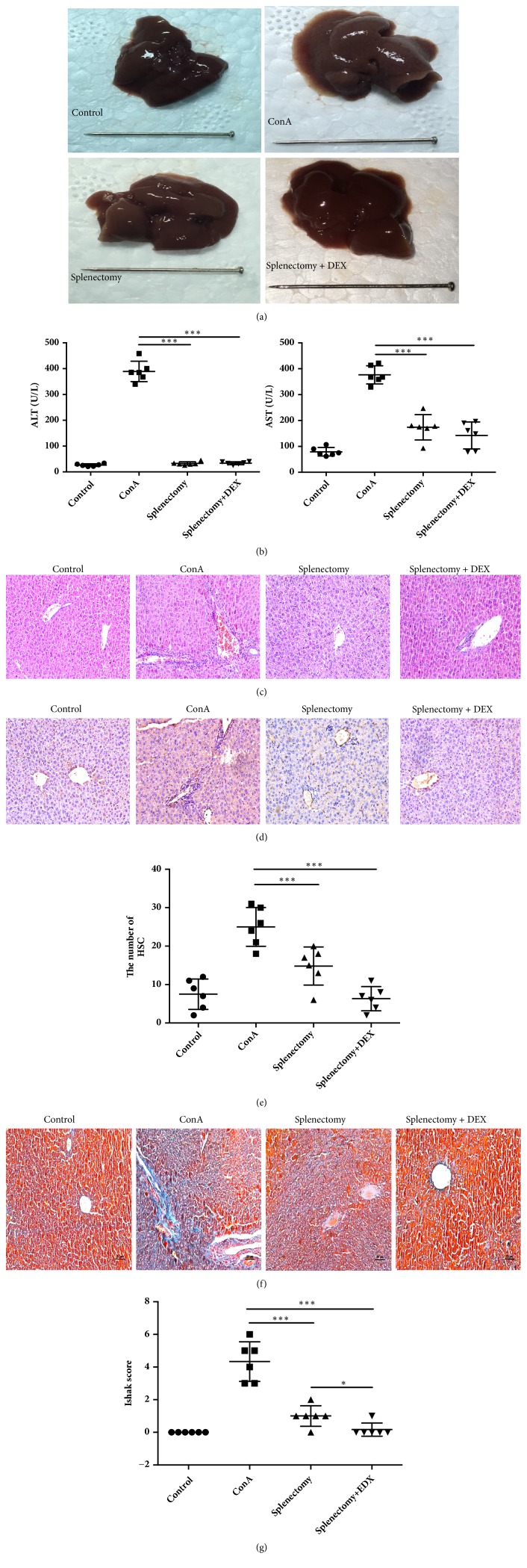
Morphologic features, histological and immunohistochemical staining (magnification ×200), and alanine (ALT) and aspartate aminotransferase (AST) levels in liver tissues from mice in four treatment groups. (a) Gross liver tissues. (b) The levels of ALT and AST detected by enzyme-linked immunosorbent assay. (c) Hematoxylin and eosin (HE) revealing inflammatory cell infiltration in the portal area. (d) Immunohistochemistry used to detect *α*-smooth muscle actin (SMA) expression in liver tissues. (e) Numbers of activated hepatic stellate cells (HSC) evaluated (immunohistochemical stain). (f) Masson trichrome stain. Blue areas indicate collagen fiber deposition in the liver tissues. (g) Ishak scores. *∗* indicates* P* < 0.05; *∗∗∗* indicates* P* < 0.001.

In the ConA model group, the hepatic surfaces were dark red and granular, whereas mice were subjected to splenectomy had smooth hepatic surfaces (Figure 2(a)). Notably, splenectomy was associated with significant decreases in serum ALT and AST levels (*P*<0.05) (Figure 2(b)). An analysis of liver tissue from the splenectomy group revealed a decrease in interface hepatitis (Figure 2(c)) and significant attenuation of liver fibrosis, as indicated by a semiquantitative analysis of *α*-SMA-positive areas (*P*<0.05) (Figures 2(d) and 2(e)), Masson-stained areas (Figure 2(f)), and Ishak scores (*P*<0.05) (Figure 2(g)). The Ishak score was lower in the DEX group relative to the splenectomy group (*P*<0.05).

### 3.3. Percentages of Macrophages/Monocytes and M2 Macrophages

**Figure 3:**
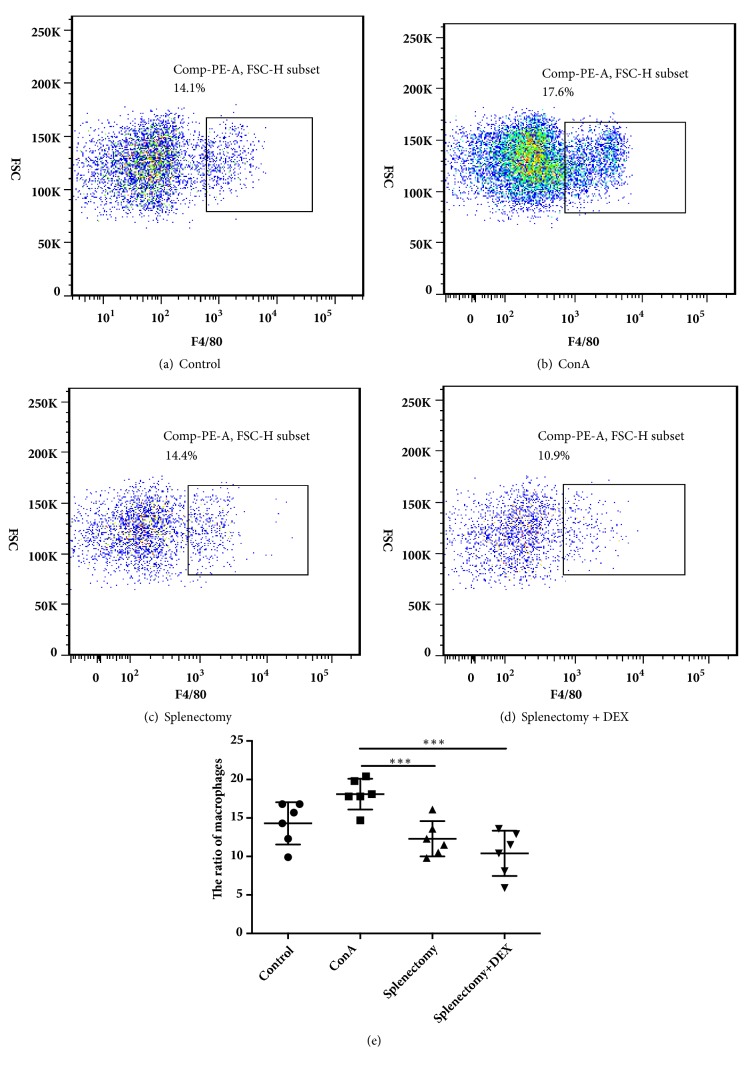
Flow cytometric analysis of monocytes from mice in (a) control, (b) ConA-treated, (c) splenectomy, and (d) splenectomy+DEX groups, and (e) the ratios of F4/80 monocytes. *∗∗∗* indicates* P* < 0.001.

**Figure 4:**
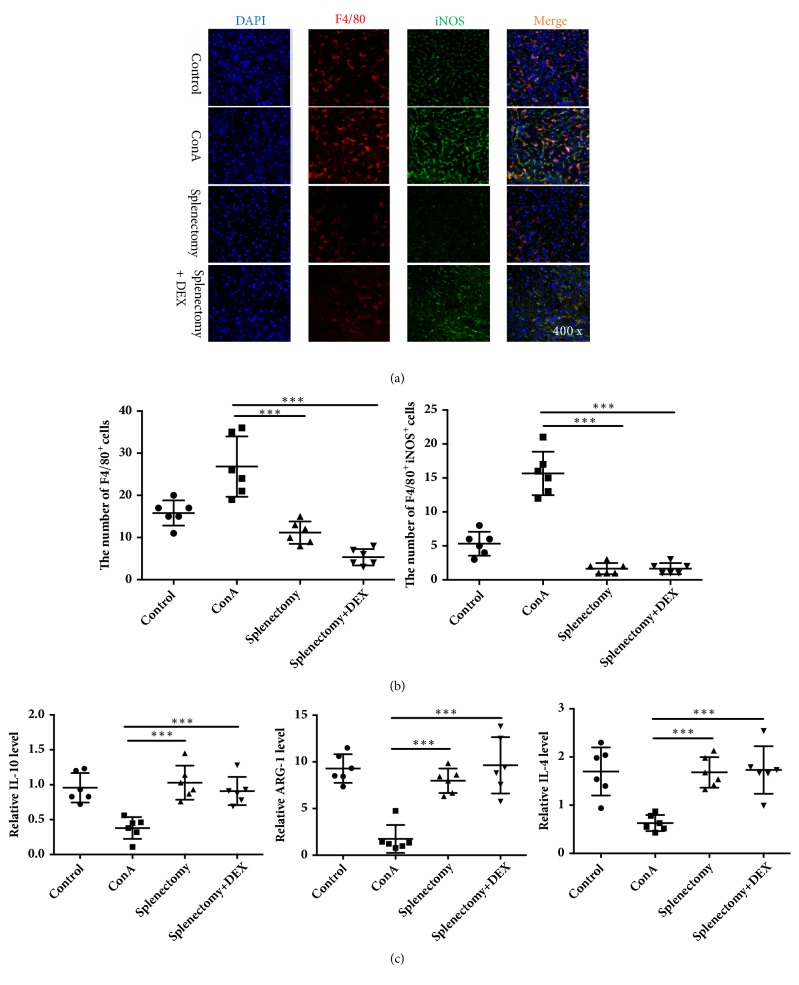
F4/80^+^ macrophages and F4/80^+^ inducible nitric oxide synthase (NOS)^+^ macrophages were detected by (a) immunofluorescent staining and (b) the ratios were calculated. The M2 macrophage-related cytokines (c) interleukin- (IL-) 10, arginase- (ARG-) 1, and IL-4 were detected by RT-PCR. ConA, concanavalin A; DEX, dexamethasone. *∗∗∗* indicates* P* < 0.001.

We used flow cytometry, immunofluorescence staining, and RT-PCR to detect changes in the numbers of monocytes in peripheral blood and liver macrophage populations. Notably, the ratio of F4/80-positive monocytes in the peripheral blood increased significantly in the ConA model group but decreased in the splenectomy group (*P*<0.05) (Figure 3). Immunofluorescence staining (Figure 4(a)) revealed increases in the percentages of F4/80-positive macrophages and M1 (F4/80^+^iNOS^+^) macrophages after ConA treatment, as well as decreases in both populations after splenectomy (*P*<0.05) (Figure 4(b)). Furthermore, RT-PCR revealed the increased expression of mRNAs encoding M2 macrophage-associated factors, such as IL-10, ARG-1, and IL-4, after splenectomy (*P*<0.05) (Figure 4(c)).

### 3.4. Splenectomy Promoted the Differentiation of *CD*11*b*^+^*Ly*6*C*^*high*^ MDSCs in the Peripheral Blood

**Figure 5:**
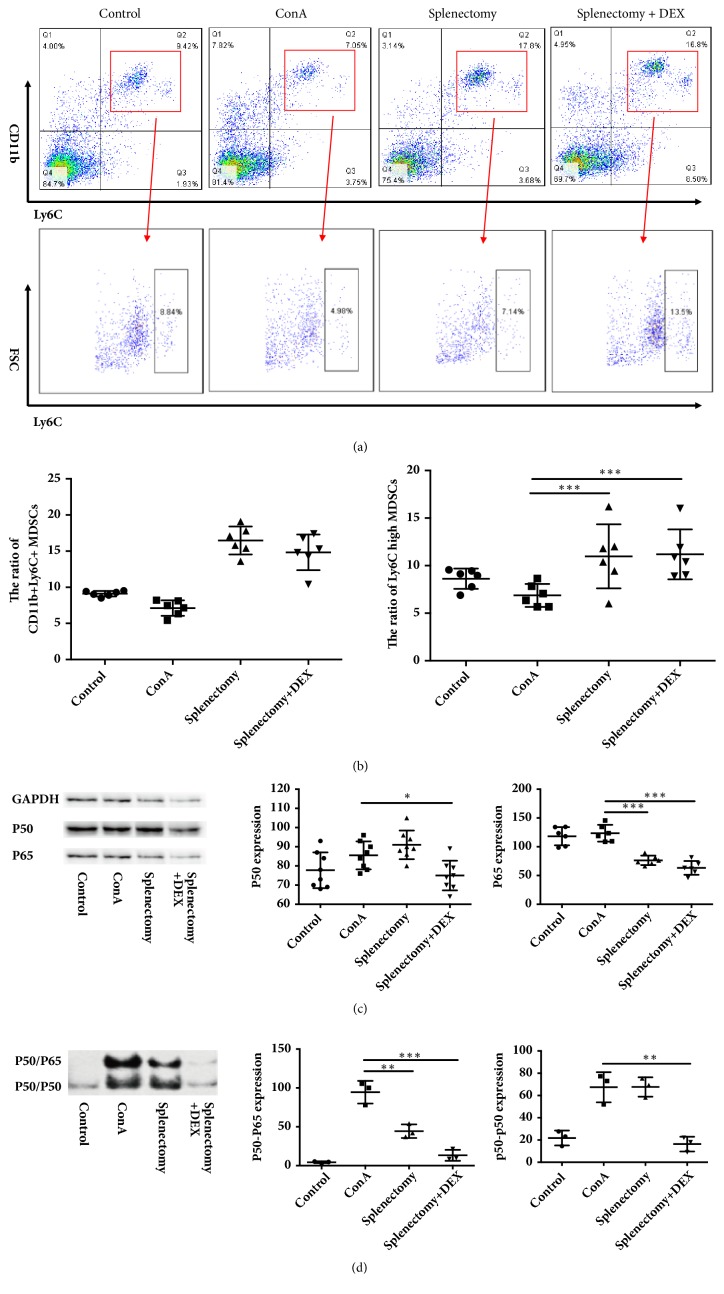
The ratios of CD11b^+^Ly6C and CD11b^+^Ly6C^high^ myeloid-derived suppressor cells (MDSCs) were detected by flow cytometry. (a) Flow cytometry analysis, (b) the ratios of CD11b^+^Ly6C and CD11b^+^Ly6C^high^ MDSCs, (c) the levels of p65 and p50, and (d) ratios of p65/p50 and p50/p50 in treatment different groups of mice. ConA, concanavalin A; DEX, dexamethasone. *∗* indicates* P* < 0.005, *∗∗* indicates* P* < 0.01, and *∗∗∗* indicates* P* < 0.001.

To evaluate the potential involvement of MDSCs in ConA-induced immune liver fibrosis, we used flow cytometry to analyze peripheral MDSCs. Notably, the proportion of CD11b^+^Ly6C^+^ MDSCs decreased after ConA treatment but increased significantly after splenectomy (*P*<0.05) (Figures 5(a) and 5(b)). We also used flow cytometry to analyze changes in the percentage of CD11b^+^Ly6C^high^ MDSCs in the peripheral blood. Here, the percentage of CD11b^+^Ly6C^high^ MDSCs decreased after ConA treatment but increased significantly after splenectomy (*P*<0.05) (Figures 5(a) and 5(b)).

### 3.5. Splenectomy Inhibited NF-*κ*B Signaling in Immune Hepatic Fibrosis

We next used Western blotting to analyze the levels of NF-*κ*B p65 and NF-*κ*B p50 proteins in the liver. The level of p65 decreased dramatically after splenectomy (*P*<0.05), whereas the expression of p50 was increased after splenectomy (*P*<0.05) (Figure 5(c)).

Finally, to analyze the DNA-binding capacity of nuclear p65, we used EMSA to measure the amounts of the NF-*κ*B p50/p65 heterodimer and p50/p50 homodimer that had bound to a specific site using a ^32^P-labeled probe containing the −178 site. A band larger than the p50 homodimer band was extracted from cells cotransfected with p50 and p65 and indicated that p50/p65 could bind to the specific site, suggesting that the levels of both p50/p65 and p50/p50 increased significantly after ConA treatment (*P*<0.05). In contrast, the level of the p50/p65 heterodimer decreased significantly after splenectomy (*P*<0.05). Furthermore, the levels of both p50/p65 and p50/p50 were significantly decreased in the DEX group (*P*<0.05) (Figure 5(d)).

## 4. Discussion

Liver cirrhosis greatly limits the use of immunosuppressants in patients with AIH [1]. Several studies have reported the potential protective effects of splenectomy liver function parameters in this patient population [2, 4]. For example, in a series of 12 patients with end-stage AIH who underwent liver transplantation, Xu et al. found that splenectomy could prevent the posttransplantation recurrence of AIH [3]. In previous studies, ConA has always been used at a dose of 15–20mg/kg body weight to induce acute liver injury in mice [26]. However, we successfully established a mouse model of immune liver fibrosis using a ConA dose of 12.5 mg/kg body weight for up to 5 weeks. In our ConA model, we observed significant increases in serum ALT and AST levels, the spleen/body weight ratio, and the tissue level of *α*-SMA. We also found that splenectomy could significantly reduce these signs of ConA-induced liver fibrosis.

Macrophages and macrophage-related factors have been reported to play an essential role in the pathogenesis of hepatic lesions and fibrosis [9–11]. Jindal et al. observed a significant increase in F4/80-positive macrophages in NASH mice and significant decreases in the expression of M1 macrophage-related markers, such as IL-10 and CXCL10 [27]. However, the polarization of M1/M2 macrophages in the context of AIH remained unclear. Interestingly, we found that the total and M1 macrophage populations were increased in AIH patients, whereas the M2 macrophage population had decreased. In our ConA-induced mouse model of liver fibrosis, we similarly observed increases in macrophages and M1 macrophages and a decrease in M2 macrophages, consistent findings in AIH patients. After splenectomy, the numbers of macrophages and M1 macrophages in our model mice decreased, while the expression of M2 macrophage-related cytokines increased. These findings suggest that M2 macrophages play an important role in suppressing liver fibrosis in AIH.

Umemura et al. reported that MDSCs exhibit pleiotropic characteristics of both M1 and M2 monocytes/macrophages [28]. In recent years, MDSCs have been identified in the context of various inflammatory and immune responses, including parasitic infections and autoimmune reactions [29, 30]. However, few studies have evaluated MDSCs in patients with AIH. In a BALB/c mouse model of ConA-induced acute immunological liver injury, Zhang et al. found that rapamycin promoted the proliferation of CD11b^+^Gr1Ly6C^high^MDSCs, which protected against immunological hepatic injury [15]. In this study, we observed a significant decrease in the frequency of CD11b^+^Ly6C^high^MDSCs after ConA treatment but a significant increase in this population after splenectomy. Therefore, we suggest that CD11b^+^Ly6C^high^MDSCs may be key inhibitors of liver inflammation in AIH. We also suggest that CD11b^+^Ly6C^high^MDSCs could potentially transform into M2 macrophages.

MDSCs can differentiate into M1 and M2 macrophages in a process that is mainly regulated by STAT1 and NF- *κ*B [31]. The NF-*κ*B signaling pathway is known to regulate macrophage-driven inflammation in autoimmune diseases, and the NF-*κ*Bp50/50 homodimer is a regulator of M2 polarization^19^. Using Western blotting, we demonstrated significant increases in the protein levels of NF-*κ*Bp50 and P65 after treatment with ConA, as well as a significant decrease in the level of NF-*κ*Bp65 after splenectomy. As intracellular NF-*κ*B can only exert transcriptional activity after binding to DNA, we also performed an EMSA and found that the expression of NF-*κ*Bp65/p50 and NF-*κ*Bp50/p50 both increased significantly after ConA treatment, whereas the expression of NF-*κ*Bp65/p50 decreased after splenectomy. Most interestingly, the expression of NF-*κ*Bp50/50 decreased significantly after splenectomy combined with DEX, whereas no significant change in this expression level was observed after splenectomy alone. We speculate that hormone therapy may inhibit NF-*κ*B signaling pathway activity, whereas splenectomy only inhibits the formation of the NF-*κ*Bp65/p50 heterodimer.

Ryutaro Maruoka et al. reported that splenectomy could prolong the effects of corticosteroids in a mouse model of AIH [4]. Specifically, these authors suggested that although corticosteroid treatment protects against AIH, it allows residual splenic failed to regulate T_FH_ cells to remain after the treatment. Few previous reports have compared splenectomy with DEX therapy. In our study, we compared splenectomy alone or combined with DEX therapy. Our results indicate that splenectomy combined with DEX led to significantly reduced liver fibrosis scores, compared with the splenectomy alone. Moreover, the NF-*κ*B signaling pathway was significantly inhibited after splenectomy combined with DEX. These results suggest that DEX and splenectomy have a synergistic effect in the treatment of ConA-induced liver fibrosis in mice. However, additional studies of the specific mechanism and long-term safety are needed.

This study provides basic information regarding the potential mechanism underlying the ability of splenectomy to inhibit liver inflammation. However, the exact mechanisms involving MDSCs, macrophages, and the NF-*κ*B signaling pathway will require further study. Future research regarding therapeutic applications should aim to understand the mechanism underlying macrophage phenotype switches* in vivo*.

**Figure 6:**
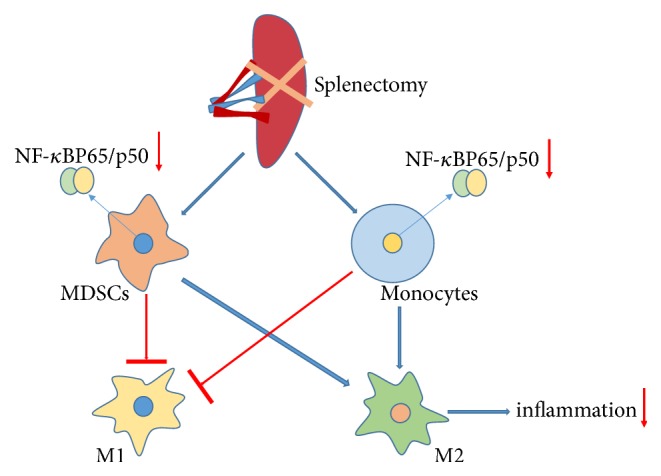
Proposed mechanisms by which splenectomy suppresses liver fibrosis in a mouse model.

In summary, we have evaluated the role of splenectomy in the observed accumulation of CD11b^+^Ly6C^high^MDSCs in a mouse model of liver fibrosis. Notably, splenectomy induced the proliferation of M2 macrophages, possibly by inhibiting activation of the NF-*κ*B signaling pathway (Figure 6). This finding suggests that MDSCs could be amplified* in vitro* and used therapeutically and indicates a new concept for the treatment of autoimmune diseases.

## Figures and Tables

**Table 1:** Oligo sequences for RT-PCR used in this study.

genes	Forward	Reverse
*β*-actin	5′-TGTGTCCGTCGTGGATCTGA-3′	5′-CCTGCTTCACCACCTTCTTGA-3′
IL-10	5′-TGGACAACATACTGCTAACCG-3′	5′-GGATCATTTCCGATAAGGCT-3′
ARG-1	5′-TGGCTTGCGAGACGTAGAC-3′	5′-GCTCAGGTGAATCGGCCTTTT-3′
IL-4	5′-CACCAGCTATGCATTGGAGA-3′	5′-TTTGGCGGTCAATGTATTTCT-3′

## Data Availability

The data used to support the findings of this study are available from the corresponding author upon request.

## Additional Points

*Key Summary*. Our research findings enabled us to newly clarify the molecular mechanism underlying the macrophage/monocyte activation and the inflammatory response in autoimmune hepatitis (AIH). We also newly detected the role of splenectomy in CD11b^+^Ly6C^high^MDSCs accumulation in a mouse model of liver fibrosis and demonstrated that splenectomy could induce the proliferation of M2 macrophages, possibly by inhibiting NF-*κ*B signaling pathway activation.
